# Integrated predictors of outcome in vertebrobasilar artery occlusion treated With endovascular therapy

**DOI:** 10.3389/fsurg.2026.1763452

**Published:** 2026-07-31

**Authors:** Hailiang Li, Yu Feng, Bo Du, Zhihan Liu, Jiuxin Gao, Deqin Geng, Yanbo Cheng

**Affiliations:** 1 Department of Neurology, The Affiliated Hospital of Xuzhou Medical University, Xuzhou, China; 2 Neurology Department, Lianyungang Second People’s Hospital, Lianyun Gang, China

**Keywords:** basilar artery occlusion, endovascular therapy, posterior circulation stroke, prognostic factors, vertebrobasilar artery occlusion

## Abstract

Endovascular therapy (EVT) for vertebrobasilar artery occlusion (VBAO) has shown encouraging results in recent randomized trials, yet functional outcomes remain highly variable despite high rates of recanalization. This discrepancy suggests the need to better understand the factors that determine whether reperfusion translates into meaningful neurological recovery. In this review, we summarize current evidence on clinical, imaging, and procedural predictors associated with prognosis following EVT for VBAO. Clinical severity and level of consciousness provide initial insight but are insufficient for reliable decision-making. Imaging—particularly the assessment of brainstem involvement, collateral circulation, and perfusion status-emerges as the most decisive predictor of outcome and an important determinant of futile vs. effective recanalization. Procedural factors, including first-pass effect, treatment timing, device strategy, and anesthesia selection, further influence the likelihood of achieving functional improvement. By integrating these multidimensional predictors, this review proposes a comprehensive framework to support more precise patient selection and guide future research aimed at improving outcomes in posterior circulation stroke.

## 1 Introduction

Acute vertebrobasilar artery occlusion is among the most devastating subtypes of ischemic stroke, with mortality reaching 35.9% even when intravenous thrombolysis is administered promptly (1). While randomized controlled trials such as MR CLEAN, ESCAPE, and SWIFT PRIME firmly established the benefit of EVT for anterior circulation large-vessel occlusion, the therapeutic advantage of EVT in posterior circulation stroke remained unclear for many years (2–4). Earlier VBAO-specific trials, including BEST and BASICS, were constrained by small sample sizes, substantial crossover between treatment arms, and heterogeneous enrollment criteria, ultimately leading to inconclusive outcomes (5, 6). The treatment landscape shifted notably with the publication of the BAOCHE and ATTENTION trials in 2022, which provided strong evidence that EVT can improve recanalization rates and functional outcomes in appropriately selected patients with VBAO. Nonetheless, an important clinical challenge remains: successful recanalization does not always translate into meaningful neurological recovery. In both BAOCHE and ATTENTION, a considerable proportion of patients did not achieve functional independence at 90 days despite technically successful reperfusion. These observations suggest the potential influence of factors such as the particular vulnerability of brainstem structures, relatively limited collateral pathways in the posterior circulation, and the inherent difficulty of accurately estimating infarct core in this anatomical territory (7, 8).

VBAO patients’ outcomes following EVT procedures show substantial variability. Various clinical, imaging, and procedural variables associated with patients’ outcomes have been identified; however, their predictive value varies considerably depending on different clinical studies (9). Furthermore, there is a lack of standardized imaging criteria used to select candidates for posterior circulation stroke interventions. While different scales have been proposed to identify patients’ eligibility based on pc-ASPECTS, collateral scores, diffusion-weighted imaging scores, and CT perfusion parameters, none of them provides satisfactory sensitivity or full anatomical coverage (10, 11).It should be emphasized that procedural factors, such as the first-pass effect, thrombectomy technique, anesthesia procedure, and treatment time, also impact patients’ outcomes but remain not fully studied yet. The absence of a unified theoretical and practical framework, which would help to estimate all possible factors influencing patients’ outcomes after EVT procedures for VBAO, may become a significant issue.

This study aims to provide a comprehensive overview of current knowledge about the role of various factors, including clinical, laboratory, imaging, and procedural, which could be applied to predict VBAO patients’ outcomes following EVT interventions. Moreover, the present study will try to develop a conceptual framework that could incorporate different aspects related to EVT interventions and help physicians make decisions concerning posterior circulation stroke.

## 2 Literature search

A structured literature search was conducted in PubMed, Web of Science, and the Wanfang database to identify studies evaluating prognostic factors associated with endovascular therapy for vertebrobasilar artery occlusion. The search covered publications from database inception to November 2025. The following keywords and their combinations were used: “vertebrobasilar artery occlusion,” “basilar artery occlusion,” “posterior circulation stroke,” “endovascular treatment,” “mechanical thrombectomy,” “prognosis,” “predictors,” and “outcomes.”

Titles and abstracts were screened for relevance, and full-text articles were reviewed when appropriate. Duplicate records were removed prior to screening. Studies were included if they involved adult patients with VBAO and evaluated clinical, imaging, laboratory, or procedural determinants of outcome after EVT. Case reports, conference abstracts, narrative descriptions without primary data, and studies lacking relevant outcome measures were excluded.Given the narrative nature of this review, formal risk-of-bias assessment and quantitative synthesis were not performed. Instead, studies were selected to reflect representative cohorts and major developments in the field, and findings were interpreted with consideration of heterogeneity in study design and patient populations.

## 3 Baseline clinical predictors

### 3.1 Neurological severity and level of consciousness

Neurological severity at presentation is an important predictor of outcome in VBAO. The initial symptom pattern often reflects the extent of brainstem involvement. In a cohort of 548 patients, Wang et al. found that individuals presenting with nonspecific posterior circulation symptoms—such as dizziness, nausea, or gait instability—were more likely to achieve favorable outcomes after EVT, whereas classic brainstem manifestations including quadriparesis, dysarthria, and impaired consciousness were associated with poorer prognosis (12). Baseline NIHSS score offers further prognostic value. Li et al. identified an NIHSS >20 as independently associated with adverse outcomes, reflecting the frequent coexistence of high clinical severity and early brainstem infarction (13). In contrast to anterior circulation stroke, posterior circulation occlusions, particularly basilar artery occlusion, may present with deceptively low NIHSS scores despite life-threatening pathology (14, 15).

Recent studies suggest that neurological severity should not be evaluated in isolation but interpreted alongside imaging findings (16, 17). While a high NIHSS score often indicates severe deficits, some patients may still benefit from endovascular treatment if collateral circulation is preserved and diffusion-weighted imaging (DWI) shows limited infarct size (18). On the other hand, patients with only mild symptoms may harbor large vessel occlusions and substantial ischemic injury, leading to poor outcomes if left untreated (19, 20). Therefore, NIHSS should not be interpreted as an isolated determinant of EVT eligibility. Patients with low NIHSS scores may still have large vessel occlusion and evolving brainstem ischemia, particularly when imaging demonstrates basilar artery thrombosis, limited infarct burden on DWI, or preserved collateral circulation. In such cases, endovascular therapy may be justified to prevent neurological deterioration.

### 3.2 Vascular risk factors

Traditional vascular risk factors also affect outcomes, although the evidence remains heterogeneous. A meta-analysis by Xun et al. (21). hypertension, diabetes, and prior stroke were associated with poorer prognosis. These associations may relate to long-standing vascular injury and reduced tolerance to ischemia. In contrast, hyperlipidemia was linked to more favorable outcomes, a pattern often attributed to the widespread use of statins rather than to lipid levels themselves, given the neuroprotective and anti-inflammatory effects of statin therapy. Atrial fibrillation and other cardiac conditions did not show consistent relationships with functional outcome, likely reflecting differences in stroke mechanisms—particularly the mix of cardioembolic and atherosclerotic etiologies—across studies.

### 3.3 Baseline functional status

Baseline functional status is an important determinant of outcome in patients undergoing EVT for VBAO. A recent meta-analysis further refined this relationship by showing that patients who were asymptomatic or without disability prior to stroke onset had the highest likelihood of favorable outcomes after EVT. Those with mild pre-stroke disability (mRS 1–2) had comparatively lower functional recovery rates than fully independent patients, yet still benefited more from EVT than from conservative therapy (22). These findings suggest that mild pre-existing disability influences prognosis but does not reduce the overall treatment benefit of EVT, supporting an inclusive approach to patient selection in this subgroup.

### 3.4 Proposed clinical risk stratification

Based on current evidence, patients presenting with marked neurological deficits—such as NIHSS scores above 20 or clear brainstem involvement—tend to fall toward the higher end of the clinical risk spectrum (23). These individuals often warrant closer imaging evaluation to assess the extent of potentially salvageable tissue. Patients with moderate symptoms lie in an intermediate range in which clinical severity alone provides limited prognostic clarity, and imaging findings, particularly infarct burden and collateral status, become more influential in estimating the likely benefit of EVT (24). At the lower end of the spectrum are patients with mild deficits, nonspecific posterior circulation symptoms, and preserved pre-stroke function. When advanced imaging does not reveal a large infarct core, these patients generally have a more favorable outlook following EVT (25). Overall, clinical presentation offers an initial framework for judging risk, but its prognostic value improves substantially when interpreted alongside imaging markers, which help refine expectations for functional recovery.

## 4 Laboratory biomarkers

### 4.1 Inflammatory markers

Inflammation is regarded as an important contributor to ischemic injury and microvascular dysfunction in posterior circulation stroke. Among inflammatory markers, the neutrophil-to-lymphocyte ratio (NLR) has been studied most frequently. Higher NLR and platelet-to-lymphocyte ratio (PLR) have been associated with poorer functional outcomes after EVT, including in patients who achieve successful reperfusion (26, 27)，Evidence, however, is not entirely consistent. A single-center study reported no clear relationship between elevated NLR and postoperative hemorrhage or mortality (28). Differences in study design, timing of blood sampling, and underlying stroke mechanisms may partly explain the inconsistent findings. Neutrophil activation can impair microvascular flow and disrupt the blood–brain barrier, which may contribute to worse outcomes in some patients. Other inflammatory markers, such as C-reactive protein (CRP) and erythrocyte sedimentation rate (ESR), have also been linked to unfavorable prognosis, although age-related variability has been reported (29). Given their limited specificity, these biomarkers may be most informative when interpreted together with clinical and imaging assessments.

### 4.2 Coagulation markers

Hypercoagulability may influence EVT outcomes through effects on thrombus composition, procedural difficulty, and re-occlusion risk. Elevated D-dimer levels have emerged as a significant biomarker of hypercoagulability and thrombus burden in patients undergoing endovascular thrombectomy for acute ischemic stroke. Recent studies demonstrate that higher preprocedural D-dimer concentrations are associated with lower rates of first-pass recanalization, increased risk of distal embolization, and worse functional outcomes (30–33). These findings suggest that D-dimer may serve not only as a marker of ongoing thrombogenesis but also as a prognostic indicator for EVT efficacy. Although fibrinogen levels and platelet activity have been proposed to influence EVT outcomes through effects on clot stability and hemorrhagic risk, direct clinical evidence remains limited. Some studies involving antiplatelet agents suggest a potential role for platelet inhibition in improving recanalization rates and reducing post-procedural complications (34, 35). However, the predictive value of fibrinogen and platelet count alone requires further investigation in well-designed clinical cohorts.

Consequently, higher inflammatory and thrombotic biomarker levels are consistently associated with worse outcomes after EVT, whereas patients with comparatively lower levels tend to demonstrate more favorable functional recovery in cohort-based stratified analyses.

## 5 Imaging predictors

Imaging plays a central role in assessing tissue viability and outcome prediction in vertebrobasilar artery occlusion (VBAO). Prognostic tools in posterior circulation stroke can be broadly grouped into three categories: structural injury scores, collateral and vascular status scores, and perfusion-based metrics. These complementary approaches are discussed below according to their pathophysiological focus.

### 5.1 Structural brainstem and infarct burden scores (pc-ASPECTS, PMI, novel-PC)

#### 5.1.1 Posterior circulation-Alberta stroke program early CT score (pc-ASPECTS)

Pc-ASPECTS is one of the most extensively evaluated imaging predictors in VBAO (36). When applied to non-contrast CT or CT angiography source images, it quantifies early ischemic changes across ten regions of the brainstem and cerebellum (37). Higher scores are generally associated with better outcomes, whereas values at or below 5–6 relate to poor prognosis and an increased likelihood of futile recanalization. Several studies have confirmed its independent predictive value, with thresholds of 6 or 7 linked to a higher probability of functional independence after EVT (38, 39). These associations support the view that early hypoattenuation corresponds to irreversible infarction or critically reduced perfusion. Importantly, pc-ASPECTS can also be applied to diffusion-weighted imaging (DWI), where it may provide greater sensitivity for detecting early posterior fossa infarction compared with NCCT (40). DWI-based pc-ASPECTS has been shown to improve lesion conspicuity in the brainstem and cerebellum, potentially enhancing prognostic accuracy in selected patients (41). However, variability in imaging timing and threshold definitions may affect inter-study consistency, and standardized cutoffs for DWI-based assessment remain less clearly established than those for CT-based applications. Despite its clinical utility, pc-ASPECTS has several notable limitations. Sensitivity in the posterior fossa is suboptimal because bone artifacts and the small size of key structures reduce the accuracy of NCCT and CTA source images. The score also omits the medulla, a region whose involvement carries important prognostic weight (42). In addition, pc-ASPECTS is a structural rather than perfusion-based measure, which limits its ability to differentiate infarct core from potentially salvageable tissue and may lead to underestimation of viable regions.

#### 5.1.2 Pons-Midbrain Index (PMI)

The Pons–Midbrain Index (PMI) is a brainstem-focused scoring system designed to quantify early ischemic changes in the pons and midbrain on CTA source images or diffusion-weighted imaging (43). Scores range from 0 to 8, with higher values indicating more extensive bilateral involvement. Several studies have reported that PMI values of 2 or lower are associated with more favorable functional outcomes after EVT for basilar artery occlusion (44, 45). Lower scores generally suggest limited brainstem injury and a greater likelihood of salvageable tissue, whereas higher scores correspond to more extensive structural damage and reduced chances of recovery. Compared with broader posterior circulation scores such as pc-ASPECTS, PMI appears to provide greater sensitivity for detecting early brainstem involvement, a feature that is often central to prognosis in VBAO.

#### 5.1.3 Novel posterior circulation score (novel-PC)

The Novel Posterior Circulation Score (Novel-PC), developed by Bofeng Bai and colleagues, is a diffusion-weighted imaging–based system designed to provide a more comprehensive assessment of ischemic burden in the posterior circulation. In clinical validation studies, lower Novel-PC scores were associated with more favorable outcomes after endovascular treatment, indicating its potential value in identifying patients with limited brainstem injury who may benefit from intervention (46). The scoring system evaluates the pons, midbrain, medulla, cerebellum, and thalamus, offering a more detailed appraisal of infarct extent and tissue viability than earlier methods. With its broad anatomical coverage and sensitivity to early brainstem involvement, Novel-PC may serve as a useful adjunct for outcome prediction and patient selection in vertebrobasilar artery occlusion.

### 5.2 Collateral and vascular Status scores (PC-CS, BATMAN)

#### 5.2.1 Posterior circulation collateral score (PC-CS)

The Posterior Circulation Collateral Score (PC-CS) is a CTA-based 10-point system that assesses collateral flow through the posterior communicating and cerebellar arteries (47). Higher scores reflect more robust collateral circulation and better compensatory perfusion of posterior circulation territories. Clinical studies have shown that patients with very low collateral scores (0–3) tend to have poor functional outcomes after EVT, and their prognosis declines rapidly with increasing time from symptom onset (48). In contrast, patients with moderate (4–5) or good (6–10) collateral supply demonstrate substantially better chances of functional improvement, with outcomes that appear less dependent on treatment delay (49). These observations suggest that collateral circulation may influence both tissue viability and the temporal dynamics of benefit from EVT. Overall, PC-CS provides complementary information to brainstem injury scores by indicating the perfusion capacity of the posterior circulation and may help identify patients who retain salvageable tissue despite vessel occlusion.

#### 5.2.2 Basilar artery on CT angiography (BATMAN) score

The Basilar Artery on CT Angiography (BATMAN) score is a CTA-based grading system that evaluates thrombus burden and collateral circulation in basilar artery occlusion (43, 50). The score incorporates assessment of basilar artery patency, posterior communicating arteries, and cerebellar branches, providing a semi-quantitative estimate of posterior circulation collateral status. Lower BATMAN scores have been consistently associated with worse functional outcomes and higher mortality following EVT (51, 52). Unlike pc-ASPECTS, which primarily reflects early ischemic structural change, BATMAN integrates both clot burden and collateral flow, offering additional insight into hemodynamic compensation. Several studies have demonstrated that BATMAN scores below 7 are associated with poor prognosis and reduced likelihood of functional independence (53). As a readily obtainable CTA-based metric, BATMAN may serve as a practical tool in emergency triage and procedural planning.

### 5.3 Perfusion-Based imaging metrics (CAPS, tmax metrics)

#### 5.3.1 Critical area perfusion score (CAPS)

The Critical Area Perfusion Score (CAPS) is a CT perfusion–based measure that assesses perfusion status in functionally important posterior circulation regions, including the cerebellum, pons, midbrain, and thalamus. Higher scores reflect better preserved perfusion within these structures. Clinical observations suggest that lower CAPS values are associated with poorer outcomes after EVT, likely reflecting more extensive impairment of tissue viability (54). By concentrating on brainstem and deep posterior circulation territories, CAPS offers information that complements structural imaging and may help identify patients who retain salvageable tissue. Given its focus on regions essential for consciousness and motor function, CAPS may serve as a useful adjunct in prognostic evaluation and in guiding EVT selection for vertebrobasilar artery occlusion.

#### 5.3.2 CT perfusion and tmax-based perfusion metrics

CT perfusion (CTP), especially when processed with automated software platforms, provides quantitative estimates of ischemic core and hypoperfused tissue (55). Although the accuracy of CTP is reduced in the posterior fossa, several perfusion-derived parameters have shown prognostic relevance in vertebrobasilar artery occlusion. Perfusion defect volume is one important marker; large perfusion deficits, typically exceeding 90 mL, have been associated with poor functional outcomes after EVT, suggesting extensive impairment of posterior circulation perfusion and limited salvageable tissue (56). Among CTP parameters, Tmax has emerged as particularly informative. A Tmax delay greater than 10 s has been repeatedly linked to unfavorable prognosis (57). Such marked delays may reflect severely compromised perfusion, microvascular dysfunction, or early irreversible injury that may not be apparent on structural imaging. Notably, severe Tmax prolongation can predict poor outcomes even in patients who subsequently achieve angiographic reperfusion, indicating its potential role in identifying tissue that is unlikely to recover (58). Taken together, perfusion defect volume and Tmax-based assessments offer useful adjunctive information for evaluating tissue viability in VBAO, particularly when identifying patients with limited opportunity for recovery or high risk of infarct progression. Because of the technical challenges inherent in imaging the posterior fossa, CTP findings are most reliable when interpreted in combination with diffusion-weighted imaging and collateral assessment.

Imaging predictors in VBAO reflect three interrelated but distinct aspects of stroke pathophysiology: structural tissue injury, collateral compensation, and perfusion delay. Structural scores such as pc-ASPECTS—whether derived from CT or diffusion-weighted imaging (DWI)—provide rapid estimation of infarct burden, while collateral and perfusion-based metrics offer complementary insight into hemodynamic reserve and potentially salvageable tissue. In clinical practice, CT-based assessments often guide immediate triage decisions due to their speed and accessibility, whereas DWI-based evaluation may improve sensitivity for early brainstem infarction when feasible. Rather than functioning as competing strategies, these tools are best interpreted in combination, particularly when selecting candidates for EVT in complex or borderline posterior circulation presentations.To help contextualize their respective advantages and limitations, the major prognostic imaging tools examined in this review are summarized in Table 1.

**Table 1:** Comparison of prognostic imaging tools in VBAO.

Score	Imaging modality	Strengths	Limitations	Suggested Use
pc-ASPECTS	NCCT/CTA-SI	Widely available; quick bedside application	Low sensitivity in posterior fossa; medulla not included; difficulty distinguishing core from penumbra	Initial screening when MRI is unavailable
PMI	CTA-SI/DWI	High specificity for brainstem ischemia; strong prognostic value	Limited anatomic scope; may miss cerebellar and thalamic lesions	Rapid assessment of brainstem injury
Novel-PC	DWI	Comprehensive evaluation of posterior circulation structures; high prognostic accuracy	Requires MRI; limited feasibility in unstable or comatose patients	Identifying viable brainstem tissue and selecting EVT candidates
PC-CS	CTA	Practical assessment of collateral flow; predicts infarct progression and outcome	Does not directly assess tissue viability; collateral scoring varies across studies	Estimating collateral support and potential therapeutic window
BATMAN	CTA	Integrates thrombus burden and collateral status; practical in emergency settings; associated with functional outcome and mortality	Does not directly quantify infarct core; collateral assessment may vary; limited sensitivity to early parenchymal injury	Assessing basilar artery patency and collateral support during initial triage and EVT planning
CAPS	CTP	Reflects perfusion integrity in critical regions; complements structural imaging	Posterior fossa susceptibility artifacts; perfusion thresholds not standardized	Evaluating perfusion reserve and ischemic progression risk
Tmax metrics	CTP	Sensitive indicator of critical perfusion delay; associated with risk of infarct growth even after recanalization	Posterior fossa imaging challenges; thresholds inconsistently applied	Adjunctive prognostication, especially when assessing risk of irreversible injury

## 6 Procedural predictors

### 6.1 First-pass effect, FPE

The first-pass effect (FPE), defined as achieving successful reperfusion with a single thrombectomy attempt, is one of the most consistent procedural predictors of favorable outcome in vertebrobasilar artery occlusion. Patients who achieve FPE are more likely to regain functional independence and tend to have lower mortality compared with those requiring multiple passes (59). Analyses in basilar artery occlusion cohorts have further demonstrated that early successful recanalization is strongly linked to good clinical outcomes and may be the only treatment-related variable independently associated with prognosis (60). The relevance of FPE is particularly notable in posterior circulation stroke, where repeated device manipulation may increase the risk of distal embolization or compromise of small perforator arteries. Given the limited ischemic tolerance of the brainstem, rapid and efficient reperfusion is especially important (61). These observations support FPE as an important procedural target and a key determinant of meaningful neurological recovery. Beyond the first pass itself, the total number of thrombectomy attempts also carries prognostic value. Patients who achieve successful reperfusion within fewer than three passes consistently show better functional outcomes and lower rates of hemorrhagic or embolic complications compared with those requiring three or more attempts (62). In the posterior circulation, each additional pass may further increase the likelihood of distal embolization, perforator injury, or endothelial trauma, mechanisms that disproportionately impact brainstem recovery. Minimizing pass number therefore appears to be an essential strategy for optimizing procedural outcomes in VBAO.

### 6.2 Device strategy: aspiration vs. Stent Retriever

Direct aspiration thrombectomy (ADAPT) and stent retriever (SR) techniques are the two principal approaches for endovascular treatment of vertebrobasilar artery occlusion. Randomized and observational studies have shown that aspiration achieves clinical outcomes comparable to those of stent retrieval (63, 64). Subsequent trials have supported this equivalence, reporting similar rates of functional independence at 90 days for both strategies (65). More recent procedural data, however, suggest that aspiration may offer certain technical advantages in posterior circulation stroke. Several analyses have reported higher reperfusion rates, shorter procedure times, and fewer distal embolic events with ADAPT compared with SR (66). A prospective study further supported these findings, noting better functional outcomes and faster recanalization in aspiration-treated patients while maintaining a similar safety profile with respect to symptomatic hemorrhage (67). Despite these observations, both aspiration and stent retriever approaches remain effective and widely used. Their overall performance appears broadly similar, and each technique may be preferable depending on individual factors such as clot characteristics, vascular anatomy, and operator experience. Device selection should therefore be individualized, with aspiration considered a strong option when rapid reperfusion and minimization of embolic risk are procedural priorities.

### 6.3 Time metrics

Timely restoration of cerebral perfusion is central to the management of ischemic stroke, and treatment delays have well-documented consequences for tissue viability (68). In vertebrobasilar artery occlusion, growing evidence indicates that the time from symptom onset to puncture (OTP) and the time from puncture to reperfusion (PTR) are important determinants of both procedural success and functional outcome (11). Prolonged OTP has been repeatedly associated with lower rates of effective recanalization and poorer prognosis, likely reflecting the rapid evolution of ischemia within brainstem structures that have limited collateral capacity. Patients who ultimately achieve favorable outcomes generally present with shorter OTP intervals, underscoring the value of rapid treatment initiation (69). Some studies suggest that the influence of time metrics may differ across treatment windows. In earlier windows, both OTP and PTR show a clear relationship with clinical outcomes, whereas in later windows the effect of OTP appears less consistent and PTR remains a more reliable predictor (70). This pattern may relate to the distinct ischemic dynamics of the posterior circulation, where irreversible brainstem injury can occur early, making the timing of procedural initiation particularly consequential.

Registry data support these findings, showing that prolonged OTP is associated with unsuccessful recanalization in VBAO. By comparison, the interval from puncture to reperfusion does not demonstrate the same consistency across cohorts, highlighting OTP as a potentially more sensitive marker of outcome. Taken together, the available evidence indicates that while efficient workflow is essential for all EVT candidates, minimizing OTP may be especially important in vertebrobasilar stroke.

### 6.4 Bridging therapy vs. Direct EVT

Intravenous thrombolysis (IVT) remains a standard component of acute ischemic stroke care and continues to be recommended for eligible patients with anterior circulation large-vessel occlusion. Evidence suggests that IVT may facilitate early recanalization and support improved outcomes in patients who later undergo mechanical thrombectomy (71–74). In posterior circulation stroke, meta-analyses indicate a possible reduction in mortality with bridging therapy; however, its effect on functional independence has been inconsistent (75). Some pooled analyses have shown benefit, whereas others have not demonstrated clear improvement, likely reflecting differences in study design, patient characteristics, clot burden, and treatment timing (76). Recent randomized trials and comparative meta-analyses have reported that direct EVT yields functional outcomes comparable to those of bridging therapy, with similar rates of successful recanalization and hemorrhagic complications (77–80). Available data also do not indicate that IVT substantially increases procedural risk in posterior circulation stroke. The variability across studies may relate to differences in imaging selection, workflow efficiency, and the limited number of trials specifically focused on vertebrobasilar occlusion.

Taken together, current evidence does not establish clear superiority of either bridging therapy or direct EVT for vertebrobasilar artery occlusion. IVT may offer benefit in selected patients, particularly those who present early or have smaller clot burdens, yet its overall impact on functional recovery remains uncertain. Further randomized trials dedicated to posterior circulation stroke are needed to determine the optimal approach. Until such data are available, treatment decisions should be individualized, considering stroke severity, clot characteristics, contraindications to IVT, and local workflow capabilities.

### 6.5 Anesthesia method

The choice between general anesthesia (GA) and conscious sedation (CS) during endovascular therapy has long been a topic of clinical interest. GA provides airway protection, patient immobility, and controlled ventilation, which may facilitate device navigation and reduce movement-related complications. However, potential drawbacks include treatment delays, hemodynamic fluctuations, and anesthesia-related risk. Evidence specific to posterior circulation stroke suggests that both approaches can achieve comparable procedural safety and functional outcomes. Case-control studies have shown no meaningful differences between GA and monitored sedation in reperfusion success or complication rates during EVT for acute posterior circulation stroke (81). A large multicenter retrospective analysis in vertebrobasilar occlusion similarly reported equivalent outcomes and safety profiles with or without GA (82). GA may be particularly useful in selected patients, especially those with markedly reduced consciousness, significant agitation, or compromised airway protection. In such cases, GA can enable safer and more controlled navigation within the posterior circulation, possibly facilitating successful reperfusion. However, some meta-analyses have suggested associations between GA and lower rates of functional independence or higher mortality compared with CS, although these findings were accompanied by substantial heterogeneity (83). Differences in patient selection, institutional workflow, and anesthesia expertise likely contribute to the variability across studies. Overall, available data do not establish a clear advantage for either GA or CS in vertebrobasilar artery occlusion. Anesthesia choice should therefore be individualized, based on the patient's neurological status, airway stability, expected procedural complexity, and the experience of the treating center. Regardless of the selected approach, maintaining hemodynamic stability and minimizing procedural delays remain essential for optimizing outcomes.

**Figure 1:**
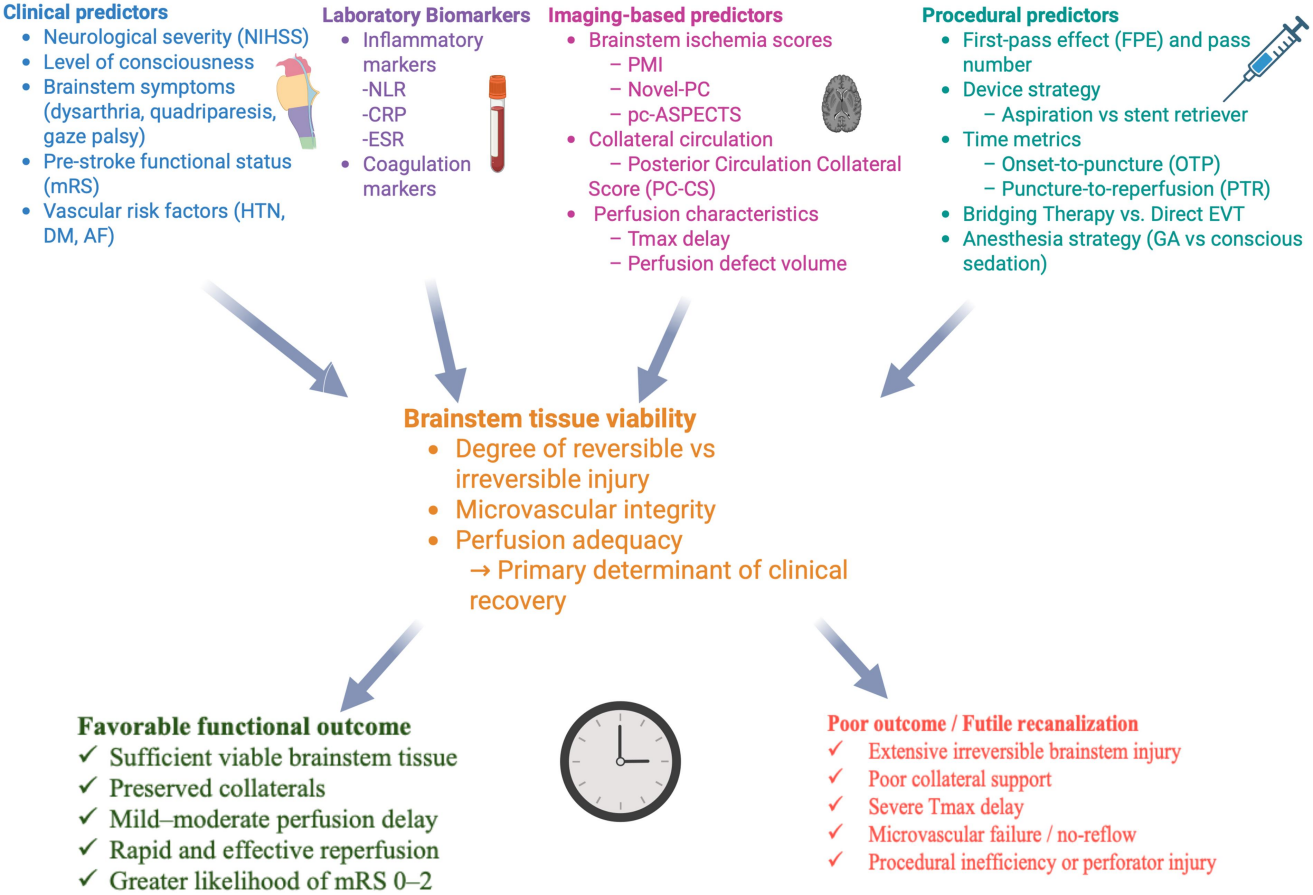
Integrated framework showing how clinical factors—including neurological status and laboratory biomarkers—imaging findings of brainstem viability and collateral flow, and procedural efficiency together determine the likelihood of meaningful recovery versus futile recanalization in vertebrobasilar artery occlusion.

Figure 1 synthesizes the prognostic determinants reviewed in Sections 3–6 and highlights brainstem tissue viability as the central determinant of functional recovery.

## 7 Discussion

Vertebrobasilar artery occlusion (VBAO) continues to show substantial variability in clinical outcomes, even with the wider use of endovascular therapy (EVT). Although EVT improves overall prognosis, many patients do not regain functional independence, indicating the importance of understanding factors that influence recovery (84).

Clinical presentation provides an early impression of disease severity but alone does not reliably predict outcome. Severe neurological deficits or reduced consciousness often correspond with early brainstem involvement, yet some patients with marked symptoms still improve when reperfusion is achieved in a timely manner. Conversely, mild symptoms do not exclude significant ischemic injury. Laboratory biomarkers may offer complementary information. Elevated inflammatory indices such as the neutrophil-to-lymphocyte ratio, platelet-to-lymphocyte ratio, and C-reactive protein have been associated with poorer outcomes in several studies, although findings remain inconsistent across cohorts. Coagulation parameters including D-dimer and fibrinogen have been linked to thrombus burden and infarct evolution, but their specific role in guiding EVT decision-making is not well established. Imaging assessments offer more direct insight into tissue viability. Brainstem-focused tools such as the Pons-Midbrain Index and the Novel-PC score help identify early ischemic injury with greater anatomical specificity. Collateral evaluations through the PC-CS further characterize the capacity to sustain perfusion during large-vessel occlusion. Perfusion imaging, particularly the presence of marked Tmax delay, has shown additional value by identifying patients who appear at higher risk for infarct progression even when vessel recanalization is later obtained. Collectively, these findings emphasize that outcomes in VBAO depend largely on the extent of brainstem injury and the availability of collateral support rather than on angiographic findings alone. Procedural considerations also influence prognosis. Faster and more effective reperfusion, especially when achieved during the first retrieval attempt, has been associated with better clinical outcomes. Aspiration techniques may reduce procedure time in certain settings, although both aspiration and stent retrievers have demonstrated broadly comparable safety and efficacy in posterior circulation stroke. Treatment timing remains a critical factor, and delays prior to arterial puncture have been linked to worse outcomes, which may relate to the limited ischemic tolerance of brainstem tissue. Regarding anesthesia modality, current evidence does not show a consistent advantage of either general anesthesia or conscious sedation, and selection is generally guided by patient condition and procedural requirements.

A recurring challenge in VBAO is the occurrence of futile recanalization, in which technically successful reperfusion is not accompanied by neurological recovery. Contributing factors described in the literature include limited collateral circulation, rapid infarct progression within the brainstem, microvascular dysfunction, and systemic inflammatory responses. Overall, available evidence supports the use of a multimodal approach when evaluating EVT candidacy in VBAO. On the basis of the evidence summarized in this review, a multidimensional framework may offer a more practical approach to patient evaluation. Such an approach incorporates neurological severity and level of consciousness, vascular risk profile and pre-stroke functional status, inflammatory and coagulation markers, imaging-based assessment of structural injury, collateral circulation, and perfusion parameters, as well as procedural elements including time metrics and reperfusion quality. Considered together, these domains relate to brainstem tissue viability and may assist in refining individualized EVT decision-making.

Further prospective and multicenter investigations are needed to better define how these variables can be integrated into clinically applicable stratification strategies.

## 8 Conclusion

Outcomes in VBAO are shaped less by vessel recanalization alone than by the underlying viability of brainstem tissue, collateral support, and the timeliness of intervention. Integrating clinical assessment with brainstem-focused imaging and procedural efficiency offers a more reliable basis for identifying patients who may benefit from EVT. Continued refinement of multimodal prediction strategies will be essential for improving patient selection and overall outcomes in posterior circulation stroke.
